# Effects of Treatment with the Hypomethylating Agent 5-aza-2′-deoxycytidine in Murine Type II Collagen-Induced Arthritis

**DOI:** 10.3390/ph12040174

**Published:** 2019-11-27

**Authors:** Maria Cristina Petralia, Emanuela Mazzon, Maria Sofia Basile, Marco Cutuli, Roberto Di Marco, Fabiola Scandurra, Andrea Saraceno, Paolo Fagone, Ferdinando Nicoletti, Katia Mangano

**Affiliations:** 1 IRCCS Centro Neurolesi Bonino Pulejo, C.da Casazza, 98124 Messina, Italy; m.cristinapetralia@gmail.com (M.C.P.); emanuela.mazzon@irccsme.it (E.M.); 2 Department of Biomedical and Biotechnological Sciences, University of Catania, 95123 Catania, Italy; sofiabasile@hotmail.it (M.S.B.); fabiolascandurra.osteopatia@gmail.com (F.S.); andreasara96@gmail.com (A.S.); paolofagone@yahoo.it (P.F.); kmangano@unict.it (K.M.); 3 Department of Medicine and Health Sciences “Vincenzo Tiberio”, University of Molise, 86100 Campobasso, Italy; marcocut@hotmail.it (M.C.); roberto.dimarco@unimol.it (R.D.M.)

**Keywords:** DAC, CIA, rheumatoid arthritis, epigenetics

## Abstract

The emerging role of epigenetics in the pathogenesis of autoimmune diseases has recently attracted much interest on the possible use of epigenetic modulators for the prevention and treatment of these diseases. In particular, we and others have shown that drugs that inhibit DNA methylation, such as azacitidine (AZA) and decitabine (DAC), already used for the treatment of acute myeloid leukemia, exert powerful beneficial effects in rodent models of type 1 diabetes, multiple sclerosis, and Guillain Barrè syndrome. Along this line of research, we have presently studied the effects of DAC in a murine model of rheumatoid arthritis induced by type II collagen and have demonstrated that DAC administration was associated with a significant amelioration of the clinical condition, along with in vivo and ex vivo modification of the immunological profile of the so-treated mice, that exhibited a diminished production of Th1 and Th17 pro-inflammatory cytokines and reduction of anti-type II collagen autoantibodies.

## 1. Introduction

Rheumatoid arthritis (RA) is an immunoinflammatory disease, probably of autoimmune origin, that is primarily characterized by chronic inflammation of the joints [1]. The histological hallmark of RA consists of symmetric synovial proliferation, cartilage destruction and bone damage that appears to be driven by the synergic action of autoreactive T cells and B cells, that produce pro-inflammatory cytokines and autoantibodies (auto-Abs) capable of amplifying the vicious circuit of cartilage destruction and inducing synovial hyperplasia [2].

It is generally accepted that, on the basis of their functions and cytokine secretory capacities, the CD4+ T helper cells (CD4+T cells) can be subdivided into at least five subsets, i.e., the pro-inflammatory Th1 and Th17 subsets, that produce the prototypical cytokines IFN-gamma, TNF-alpha and IL-17 [3,4], and the anti-inflammatory Th2 and Th3 cells, that produce the anti-inflammatory cytokines IL-4, IL-10, and TGF-beta [5,6,7]. The role of the Th9 cells is less well characterized, but increasing evidence indicates that these cells may also be implicated in the pathogenesis of autoimmune disorders [8].

Macrophages can also be subdivided into pro-inflammatory M1 and anti-inflammatory M2 macrophages, that are characterized by the production of the pro-inflammatory cytokines TNF-alpha, IL-6, IL-12, IL-18, and IL-23, and of the anti-inflammatory cytokine IL-10, respectively [9,10]. Both preclinical and clinical evidence coming from rodent models of RA and RA patients indicate that the CD4+T cell belonging to the Th1 and Th17 subsets, along with M1 macrophages, play a key role in the development and maintenance of RA, as opposed to anti-inflammatory Th2 and Th3 cells, and M2 macrophages that seem to play a protective role on the course of the disease [11].

That the altered balance between pro- and anti-inflammatory CD4+T cells and macrophages may play a key role in the cell mediated arthritogenic events concurs with clinical data showing that specific inhibitors of TNF-alpha, IL-1, and IL-6 exhibit clear-cut beneficial effects in patients with RA, and are approved as biological disease modifying antirheumatic drugs (DMARDs) for the treatment of the disease. Rituximab and abatacept, that target respectively the CD20 antigen on B cells and the CD80/86 costimulation pathway, are also approved as DMARDs in RA. In particular, the efficacy of Rituximab in RA patients underlines the pathogenetic relevance of B cells in the pathogenesis of this disease and indicates that the combined action of autoreactive Th1 and Th17 cells and B cells is implicated in the development and maintenance of, at least, certain forms of the disease [12]. More recent Phase II studies have also suggested a beneficial effect of IL-12/IL-23 and IL-17 inhibitors in RA patients [12].

Usually, these biological DMARDs are associated with chemical DMARDs that include glucocorticoids, non-steroidal anti-inflammatory drugs (NSAIDs), and conventional disease-modifying antirheumatic drug (cDMARD) [13]. More recently approved targeted synthetic DMARDs include the Janus kinases (JAKs)inhibitors Tofacitinib (Jak-1, Jak-2, Jak-3) and baricitinib (Jak1, JAK2) [12].

However, in spite of these multiple treatments, there still remains a significant proportion of RA patients who are either de novo poor responsive to biological DMARDs or acquire resistance due to the production of neutralizing anti-drug antibodies (ADAs) [14]. The lack of homogeneous response to treatment might be attributed to the heterogeneity of the disease [15]. In addition, the chemical DMRADs possess several side effects that limit their prolonged use. The above clearly indicates that there is urgent need to develop new drugs for the treatment of RA. In addition, an area of particular therapeutic need is represented from those patients that simultaneously suffer from RA with the immunoinflammatory demyelinating disease multiple sclerosis (MS). In fact, RA and MS are known to occur more frequently in the same patients [16]. This poses an important therapeutic challenge because, although being both recognized as autoimmune diseases, they show opposite therapeutic responses to DMARDs, with the TNF-inhibitors used for RA that are known to exacerbate the course of MS or provoke demyelination [17,18], while IFN-beta used for MS has a controversial role in RA pathogenesis and has been suggested to be capable of provoking some cases of iatrogenic RA [19,20].

Decitabine (5-aza-2′-deoxycytidine, Dacogen, DAC) is a hypomethylating agent in use for the treatment of myelodysplastic syndromes [21]. As an inhibitor of DNA methyltransferases (DNMTs), it induces demethylation and reactivation of silenced genes [22]. Previous studies by ourselves and others have shown that treatment with DAC ameliorates the course of rodent models of autoimmune diseases, such as type 1 diabetes, MS, and Guillain Barrè syndrome (GBS), likely by inducing the upregulation of FoxP3 expression and induction of regulatory T cells [23,24,25,26,27]. In particular, independent studies have concordantly proven the beneficial effects of DAC in different rodent models of experimental autoimmune encephalomyelitis (EAE) [24,25,26], that serves as a preclinical model of MS. DAC apparently acts through an immunopharmacological mode of action that entails an increase in the number of regulatory T cells, and the reduction in the proportion of Th1 and Th17 cells [23,24,25,26,27,28]. Since the defective number and function of regulatory T cells (Tregs) and increased number of Th1 and Th17 responses have been described in RA, and are thought to play a pathogenetic relevance in the induction and progression of the disease [29], we hypothesized that this emerging mode of action of DAC in EAE could also be beneficial in RA. To prove this, we have presently evaluated the effects of prophylactic and therapeutic treatment with DAC in a murine model of RA, that can be induced in susceptible strain of mice via immunization with type II collagen (CII). The data demonstrate that regardless of its regime schedule, DAC exerted significant beneficial effects on the course of murine type II Collagen-Induced Arthritis (CIA), that are associated to profound in vitro and ex vivo immunopharmacological modifications.

## 2. Results

### 2.1. In Silico Study: Prediction of Autoimmune Disease Potentially Targeted by DAC Treatment

In order to profile the transcriptomic changes induced in vivo by the administration of DAC, we interrogated the GSE72686 dataset. We identified 694 and 1289 significantly upregulated and downregulated genes, respectively, associated with DAC treatment. Anti-signature enrichment of DAC profile among autoimmune diseases was performed by using the EnrichR database. Several disorders were identified (Table 1), with the most significantly enriched being allergic contact dermatitis, ankylosing spondylitis, asthma, multiple sclerosis, rheumatoid arthritis, and ulcerative colitis (*p* < 0.00001) (Table 1). On the other hand, no significance was observed for dermatomyositis, non-systemic juvenile idiopathic arthritis, Sjogren’s syndrome, psoriasis, and systemic lupus erythematosus (Table 1).

### 2.2. Animal Study

#### 2.2.1. Toxicity

The treatment with DAC appeared to be well tolerated from the mice as judged by their clinical behavior and body weight variation when compared to that of vehicle-treated mice (data not shown).

#### 2.2.2. Effect of Late Prophylactic Treatment with DAC on the Arthritic Score and on Paw Thickness

**Figure 1:**
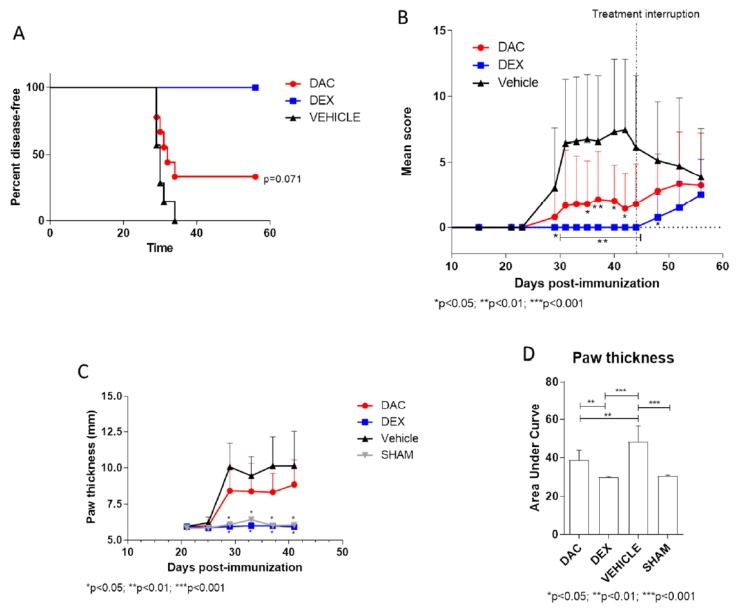
Disease incidence (**A**), clinical course (**B**), and paw thickness (**C**) in Collagen-Induced Arthritis (CIA)- mice treated in late prophylactic regimen with either decitabine (DAC), Dexamethasone (Dex), or vehicle. (**D**) Area under the curve of paw thickness measured during the entire treatment period, in CIA affected mice.

In the group of vehicle-treated mice, a gradual increase in the arthritic scores and in the thickness of the paws was observed starting from day 23 post-immunization (Figure 1A–D). The peak of the disease was reached at day 31 post-immunization, while at day 42, mice started to show disease remission (Figure 1A). The late prophylactic treatment of mice (starting on day 15 post-immunization) with DAC exerted a protective effect (Figure 1A–D), inducing a significant reduction of clinical score at day 31 (*p* < 0.05) and 35 (*p* < 0.01) and from day 40 to 42 (*p* < 0.05) and a significant reduction of cumulative paw thickness compared to vehicle-treated mice (*p* < 0.01) (Figure 1D). After the interruption of treatment at day 44, the groups of mice treated with either DAC or the positive control drug, Dexamethasone (Dex), started to show an exacerbation of their clinical conditions (Figure 1B). Treatment with Dex significantly reduced the severity of the disease (*p* < 0.05 on day 29 and *p* < 0.01 from day 31 to day 44) (Figure 1), and also significantly reduced its incidence, as compared to vehicle-treated mice (*p* < 0.01) (Figure 1A).

#### 2.2.3. Effect of Therapeutic Treatment with DAC on the Arthritic Score and on Paws Thickness

**Figure 2:**
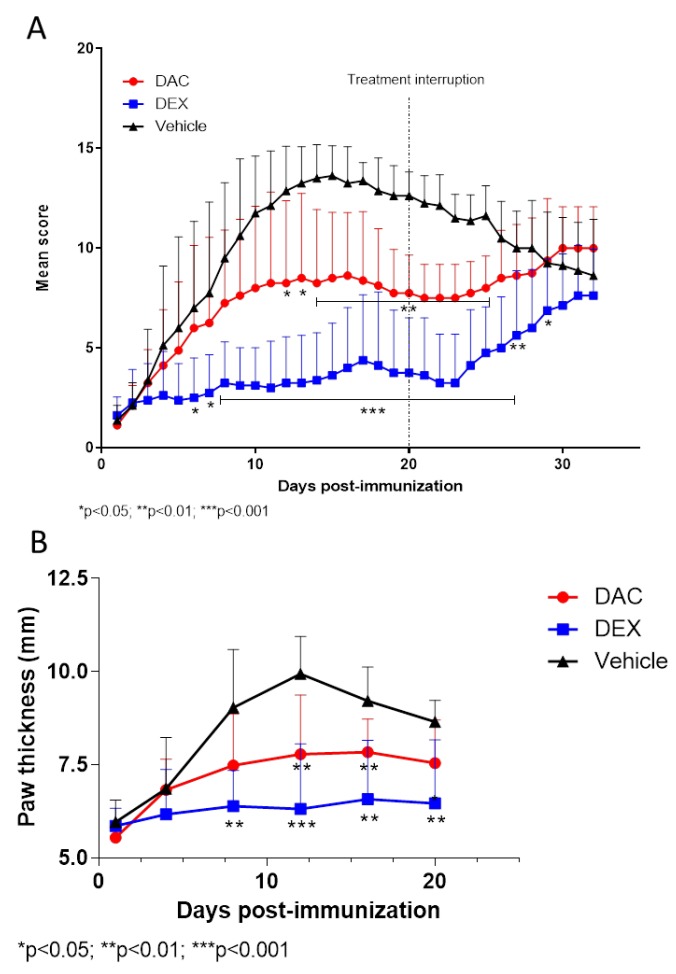
Clinical course (**A**) and paw thickness (**B**) in CIA-induced mice treated in therapeutic regimen with either DAC, Dex, or vehicle.

As expected, starting from 3 to 4 days after the second boosting, clinical signs of arthritis became observable in the mice that were equally distributed in the different groups. Mice showing a score ≥1 started the treatment. As expected, the mice treated with the vehicle exhibited a progressive increase in the arthritic scores (Figure 2A) accompanied by increased thickness of paws (Figure 2B). The treatment with DAC afforded a significant reduction of the arthritis score from day 12 to 25 (*p* < 0.05 on days 12–14 and *p* < 0.01 on days 15–25) and a significant reduction of paw thickness from day 12 compared to vehicle treated mice (*p* < 0.05). As expected, strong and significant effects were observed with the positive control drug Dex that reduced the clinical score from day 6 to day 28 (*p* < 0.05 on days 6–7, *p* < 0.001 on days 8–26, *p* < 0.01 on day 27, and *p* < 0.05 on day 27). After the interruption of the treatment, the mice were observed for additional 12 days. During this follow up period, the mice treated with either of the drugs began to exhibit an exacerbation of their clinical conditions (Figure 2A)

#### 2.2.4. Effects of DAC on Serum Anti-CII Antibodies

**Figure 3:**
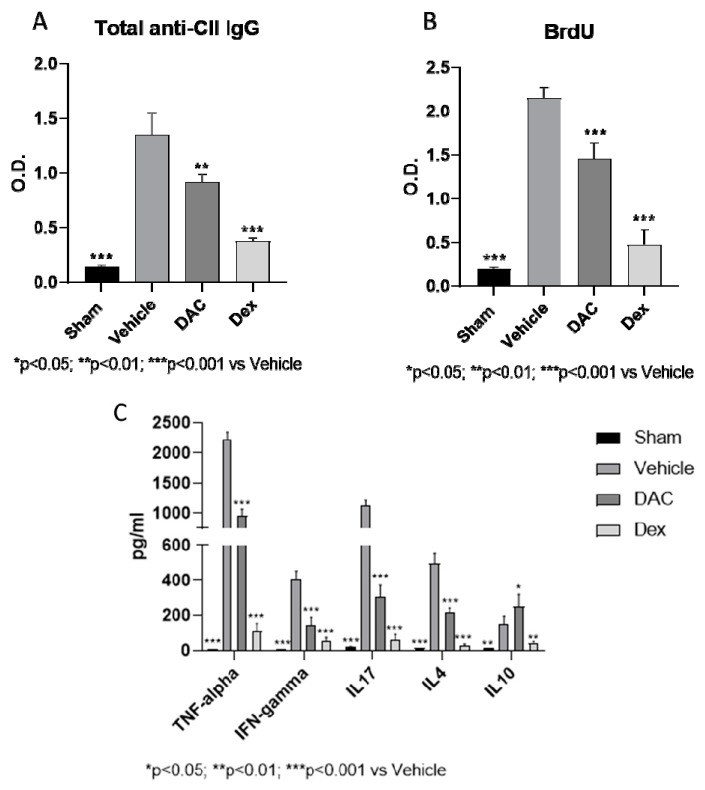
Ex vivo evaluation of total anti- type II collagen (CII) IgG (**A**), antigen-specific proliferation (**B**), and cytokine production (**C**) in splenocytes isolated from CIA-affected mice treated in prophylactic regime with vehicle, DAC, or Dex. O.D.—optical density.

Five additional mice from each group treated under late prophylactic regimen were sacrificed at the end of the treatment and blood was collected for the detection of anti-collagen type II total IgG antibodies by ELISA. The levels of these antibodies increased in the vehicle treated mice and were significantly reduced in the mice treated with DAC and Dex (Figure 3A).

#### 2.2.5. DAC Profoundly Modulated Ex Vivo Cytokine Secretion from the Spleens during Type II CIA

To gain insight into the immunopharmacological mode of action of DAC, we next examined the influence of the treatment with this drug on the cytokine secretory capacity of the spleens. To this aim, we examined the ex vivo response of splenocytes to re-stimulation with CII. A significant increased proliferation (Figure 3B) and TNF-alpha, IFN-gamma, IL-17, IL-4, and IL-10 secretion (Figure 3C) was observed in the splenocytes of vehicle treated mice compared to sham mice, indicating an acquired and autoantigen specific immunological response to CII immunization. The treatment with DAC induced a significant ex vivo reduction of this proliferation (*p* < 0.001) (Figure 3B) and an inhibition of both the Th1 and Th17 proinflammatory cytokines, TNF-alpha, IFN-gamma, IL17, as well as, the type 2 cytokine IL-4 (*p* < 0.001) (Figure 3C). On the other hand, an upregulation of IL-10 was observed for the splenocytes from DAC-treated mice as compared to vehicle-treated mice (Figure 3C). The treatment with the positive control compound Dex induced a strong reduction of the proliferative response to CII and of all the tested cytokines (*p* < 0.001).

#### 2.2.6. Effect of DAC Treatment on CIA Morphological Changes

**Figure 4:**
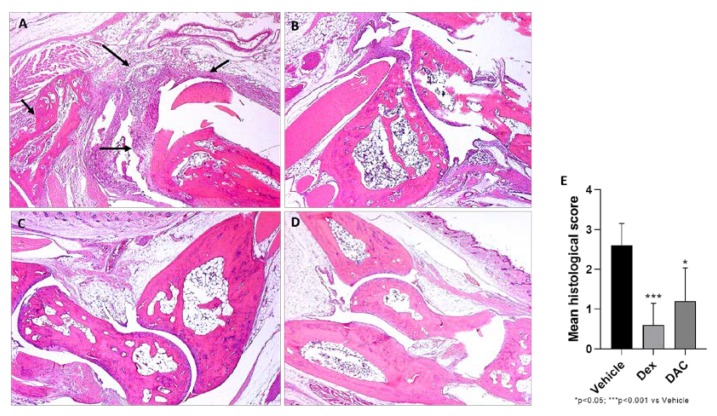
Effect of DAC treatment on CIA morphological changes. Representative articular sections hematoxylin/eosin stained, examined by optical microscopy. (**A**) Vehicle treated mice: severe arthritis with complete loss of the hyaline cartilage matrix. Severe infiltration of inflammatory granulocytes in the subchondral bone (arrows); (**B**) DAC treated mice: few inflammatory infiltrate and cartilage preservation. No signs of pathology in Dex treated mice (**C**) and Sham mice (**D**); mean histological score (**E**).

The joints of the vehicle treated group showed severe arthritis, with complete loss of the hyaline cartilage matrix that exposes the subchondral bone to the intra-articular space. In adjacent areas, severe infiltration of inflammatory granulocytes was found in the subchondral bone. Synovial hyperplasia was also detected (Figure 4). The mice treated with DAC exhibited a significant reduction of the histological score (*p* < 0.05) (Figure 4). In particular, the inflammatory infiltrate was reduced and the cartilage was preserved (Figure 4). There were no signs of pathology in the sham mice and in the dex treated mice.

## 3. Discussion

This study demonstrates, for the first time, that the hypomethylating agent DAC powerfully ameliorates the course of murine type II CIA regardless of whether it is administered upon a late prophylactic regime or at disease onset to mice with established signs of arthritis. As previously demonstrated in the EAE model, the protective effect was however short-lasting and required prolonged treatment with the drug to be maintained. In fact, exacerbation or recurrence of arthritis was observed in both groups of mice treated either prophylactically or therapeutically within 2–3 weeks after drug withdrawal.

The beneficial clinical effect was associated with reduced levels of anti-type II CIA auto-Abs and the ex vivo and in vitro inhibition of polarization of CD4+Th cells toward the Th1 and Th17 phenotypes. This is consistent with previous studies by ourselves and others demonstrating that DAC treatment inhibited the polarization of Th1 and Th17 cells in murine EAE [24,26]. However, the suppressive effect of DAC did not seem specific for Th1 and Th17 cell products, as DAC also suppressed the secretion of the prototypical type 2 cytokine, IL-4.

The mice treated prophylactically and therapeutically with DAC also exhibited lower serum titers of anti-type II collagen auto-Abs as compared to controls. This indicates that DAC may have suppressed the autoreactive function of B lymphocytes either through a direct action on these cells or via reduced activity of Th1 and Th17 cells. So far, to the best of our knowledge, no data is available in the literature on the effects of DAC on B cell function, and we are currently prioritizing this area of research. This is also of particular relevance as B cells are being recognized as important effector cells in autoimmune diseases, such as RA and MS. As discussed above, certain forms of both diseases benefit from the therapeutic use of biological drugs targeting, specifically, B cell number and function, such as rituximab and ocrelizumab [12,30].

The present data on the beneficial effects of DAC in prevention and treatment of type II CIA propels studies on the possible use of this drug in RA patients that are poorly responsive to standard of care treatment. In addition, the remarkable in vivo anti-arthritogenic potential of DAC is supported by recent evidence demonstrating its clear-cut beneficial effects in proteoglycan-induced arthritis in mice [31].

In addition to intuitively propose the use of DAC in RA patients, the simultaneous and repeatedly demonstrated efficacy of DAC in EAE [24,25,26] suggests that DAC may possess an immunopharmacological profile capable of efficiently modulating both arthritogenic and encephalytogenic responses operating in RA and MS, respectively.

This is also consistent with our present in vitro and ex vivo studies where DAC inhibited the autoantigen induced proliferation of T cells from type II CIA-affected mice, as well as the polarization toward Th1 and Th17 cells, in a manner similar to what has been shown in murine EAE [26]. If human RA and MS are also equally responsive to DAC, it could be envisaged that this drug may have the peculiar and important feature to be used as single therapeutic option for those patients that simultaneously suffer from the two diseases. This may be of clinical and therapeutic relevance as MS and RA tend to be more frequently associated in the same patient [16] and TNF-inhibitors used for RA may exacerbate or induce de novo MS, while IFN-beta has been suspected to provoke RA.

Hence, the use of a single drug for patients with both forms of the disease could reduce side effects of multiple combined treatments and prevent the development of unwanted and disease-specific side effects. In addition, the use of one single drug could allow effective cost reduction of the treatment.

Initial phase II proof-of-concept trials in RA and MS patients and in patients with simultaneous RA and MS may be easily achievable as DAC is already in the clinical setting for the treatment of cancer patients and its repurposing in RA and other autoimmune diseases may be attainable with relative ease and rapidity.

One issue arising with the prolonged use of DAC in patients with chronic diseases such as RA, MS, or both, is the lack of oral availability of DAC, that requires its infusion to the patients. Nonetheless, an analogue of DAC, named Zebularine, has been characterized and it is active per os [32,33]. However, although Zebularine has been show to downregulate autoimmune responses in experimental autoimmune uveitis [34], it has not reached the clinical development phase and complete preclinical development is required to be tested in humans.

We have not presently investigated whether, as in type 1 diabetes in Non-obese diabetic (NOD) mice [23] and rodent EAE [24,25,26] and experimental autoimmune neuritis (EAN) [27], DAC also increased the frequency and/or the function of regulatory T cells. This is likely to have occurred, as it is a known demonstrated immunopharmacological mode of action of DAC and, if so, this may have further contributed to the beneficial effects of DAC. Indeed, a defective number and function of Tregs has been described in rodent type II CIA [35] and RA patients [29]. Since induction of Tregs via hypo-methylating action on the Foxp3 gene that clusters regulatory T cells is a well described pharmacological property of DAC [23,24,25,26], we feel it is likely that this event has also occurred in this study and that this has contributed to the beneficial effects of DAC to the prevention and cure of the disease.

It should however be noticed that, regardless of the specific anti-arthritogenic pathways affected by DAC, the efficacy has been short lasting, as treatment interruption was associated with recurrence and/or exacerbation of type II CIA. This is consistent with our previous observation in EAE [26] and EAN [27], and indicates that prolonged treatment with DAC may be needed if these findings will be translated into the clinical setting.

It also remains to be demonstrated at which extent the hypomethylating effects of DAC might mediate its immunomodulatory effects in murine type II CIA. We have decided to focus this aspect in future studies, that will evaluate the emerging role of epigenetics in RA and its rodent models [36] and the support for the use of epigenetic modulators in the setting of RA and other autoimmune diseases [37,38,39].

It is however, worth mentioning that several dysregulated epigenetic mechanisms, entailing both hypo- and hyper-methylation of several genes, have been demonstrated to operate in RA [40,41,42,43]. These include alterations in DNA methylation, histone modifications, and microRNA expression. These changes in the epigenome influence key inflammatory and matrix-degrading pathways and are suspected to play a major role in the pathogenesis of autoimmune diseases [40,41,42,43]. Defining to what extent hypo-methylation will be universally beneficial in RA and how it will impact the function of hyper-methylated genes potentially related to the pathogenesis of the disease remains to be studied [44].

Nonetheless, the potential pathogenetic role of hyper-methylation has been recently demonstrated, as hyper-methylation of MEG3 (Maternally Expressed 3) gene, with consequent hyperexpression of nucleotide oligomerization domain (NOD)-like receptors 5 (NLRC5), that plays a role in immunoinflammatory and autoimmune responses, has been observed both in Complete Freund’s Adjuvant (CFA)-induced synovial tissues in rodent RA and fibroblast-like synoviocytes and the effects were counteracted by DAC in vitro [45]. It has also been shown that DNA hyper-methylation of secreted frizzled related protein (SFRP2) influences the pathology of RA through the canonical Wnt signaling in model rats [46]. On the basis of these findings, DAC could exert important “non-immunomodulatory” properties in RA also via hypo-methylating effects and modulation of epigenetic abnormalities.

In light of the clear-cut beneficial effects of DAC in animal models of autoimmune diseases, we have performed in this study, an in silico analysis for the identification of autoimmune diseases potentially treatable with DAC. On the one hand, our in silico analysis predicts the efficacy of DAC in certain immunoinflammatory and autoimmune diseases including allergic contact dermatitis, ankylosing spondylitis, and asthma that have not yet been experimentally proven. On the other hand, and further corroborating the in vivo studies, some of the identified diseases, including MS and ulcerative colitis, have already been found to be ameliorated by DAC, in preclinical models [23,47]. In addition, the in silico analysis revealed that systemic lupus erythematosus may not benefit from DAC treatment, in accordance with previous literature demonstrating that DAC could induce lupus-like complications [48,49]. Overall, combination of in silico and in vivo data strongly warrants clinical proof of concept studies to evaluate the efficacy of DAC in patients suffering from these autoimmune diseases, including RA and MS and, we feel of particular relevance, those forms that occur simultaneously.

## 4. Materials and Methods

### 4.1. In Silico Analysis

DNA microarray analysis is a widely used bioinformatic approach that allows to identify pathogenetic mechanisms and possible therapeutic targets for existing drugs, that may be repurposed for different indications. DNA microarrays have been employed in several preclinical and clinical studies of immunoinflammatory and autoimmune diseases [50,51,52,53,54,55,56], cancers [57,58,59,60,61,62,63], and neuropshychiatric disorders [64], and have led to the identification of novel and tailored therapeutic approaches [65,66,67,68,69]. In order to profile the transcriptomic changes of DAC in human immune cells, the publicly available GSE72686 dataset was used. GSE72686 included data from PBMCs of solid tumor patients who were given DAC therapy 10 mg per day for 5 days/28 day cycle. Blood samples were collected before and after 2 cycles of decitabine therapy. The Affymetrix Human Gene Expression Array was used to generate the microarray. Raw data were preprocessed by Robust Multiarray Averaging (RMA) method, implemented in the Babelomics 5 web-based utility (http://babelomics.bioinfo.cipf.es/), and identification of differentially expressed genes (DEGs) was performed by linear models for microarray analysis (LIMMA) method using the MultiEsperimentViewer software. Genes with a false discovery rate (FDR) < 0.1 and a │Fold Change│> 2 were considered as DEGs. Anti-signature enrichment of DAC transcriptomic profile was performed using EnrichR database (https://amp.pharm.mssm.edu/Enrichr/). Fisher’s Inverse Chi Square test was used to collapse adjusted *p* values from the enrichment analysis, using the following formula:

S = -ln∑^n^_i=0_ (p_i_) (1)

which follows a χ2 distribution with 2n degrees of freedom under the null hypothesis.

### 4.2. In Vivo Study

#### 4.2.1. Animals

Seven week old male DBA1 mice (Harlan Laboratories, San Pietro al Natisone, Udine, Italy) were used for the study. Mice were housed in a controlled environment and provided with standard rodent diet and water. Animal care followed local regulations on the protection of animals used for experimental and other scientific purposes (Directive 86/609/EEC, enforced by the Italian D.L. No. 116 of 27 January 1992). Animal studies were firstly approved by a local ethics Committee and then by the Ministry of Health.

#### 4.2.2. Induction of CIA

Bovine type II Collagen (Chondrex, Redmond WA, USA) was dissolved at 2 mg/mL in 0.05 M acetic acid by gently stirring overnight at 4 °C. Complete adjuvant Freund (CFA) was prepared by adding Mycobacterium tuberculosis H37Ra (Difco, Detroit, MI, USA) at a concentration of 2 mg/mL to incomplete Freund adjuvant (IFA, Sigma Aldrich, Milano, Italy). Before injection CII was emulsified with an equal volume of CFA. To induce CIA, the mice were injected intradermally at the base of the tail with 100 µL of an emulsion containing 100 µg of CII and 100 µg of Mycobacterium tuberculosis. On day 21, the second booster of CII in IFA was administered. Both intradermal administrations of collagen have been performed with the application of EMLA anesthetic cream.

#### 4.2.3. Drugs

Decitabine (DAC) (Sigma, Milan, Italy) was dissolved in dimethyl sulfoxide (Sigma, Milan, Italy) as a 2 mg/mL solution and diluted with water before injection as a 0.01 mg/mL. Dexamethasone (Soldesam, 4 mg/mL solution for injection; Laboratorio Farmacologico Milanese, Milano, Italy, purchased from a local pharmacy) was chosen as positive control drug for its anti-inflammatory effects that justify its use in the human arthritis, in addition, in our previous study, [70] the treatment with Dex in the collagen induced arthritis induced a significant amelioration of clinical symptoms and histological scores. Dexamethasone was prepared at a concentration of 0.03 mg/mL, diluting it with water for injections. The negative control group was treated with sterile water for injections. DAC and the reference drug, dexamethasone, were administered daily by intraperitoneal injection at doses of 0.1 mg/Kg and 0.3 mg/Kg, respectively. The drugs and the vehicle were administered to the mouse in a final volume of 200 μL/mouse. A group of healthy animals (sham) was used as a standard for body weight and paw thickness.

#### 4.2.4. Treatment Regimens

For the prophylactic part of the study DAC, Dexamethasone and vehicle were administered starting on day 15 after the first immunization (late prophylactic regimen) until day 44. After the interruption of treatment, mice were observed for further 12 days, to evaluate the effect of the test drug during the follow-up period. Each group of mice was composed of 8 mice, and five additional mice for each group were sacrificed at the end of the treatment, for spleen and blood collection, and for histopathological evaluation.

**Figure 5:**
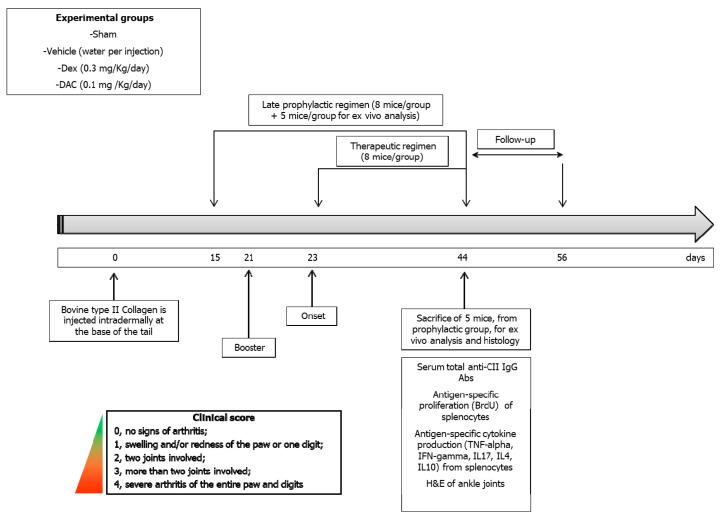
Experimental study plan.

For the therapeutic part of the study, two groups of mice were treated respectively with DAC at the dose of 0.1 mg/Kg and Dexamethasone at the dose of 0.3 mg/Kg starting within 2 days after the mice developed an arthritic score ≥ 1. The mice were randomly divided into different strata according to body weight and clinical score and, within each stratum, the animals were randomly allocated to DAC, Dexamethasone, and vehicle treatment. The treatment was continued for 20 consecutive days. After the interruption of the treatment, the mice were observed for additional 12 days, to evaluate the effect of the test drugs during the follow-up period (Figure 5). Each group of mice was composed of 8 mice.

#### 4.2.5. Primary Endpoints

##### Body Weight

The mice were weighed the day of the first immunization and three times a week starting from the second booster (day 21) until the end of the study.

##### Arthritic Score

Mice were monitored daily for the evaluation of arthritic signs based on a system that assigns a score of 0 to a maximum of 4 for each paw: 0, no signs of arthritis; 1, swelling and/or redness of the paw or of a terminal segment of the paw; 2, involvement of two joints; 3, involvement of at least three joints; 4, severe arthritis of the entire paw and of a terminal segment of the paws.

##### Assessment of Paws Thickness

Clinical severity was also determined by evaluation, twice a week, of paw thickness of both front and hind paws using a thickness gauge. An index was calculated for each mouse by summing the thickness for the individual paws.

### 4.3. Ex Vivo Studies

In the groups of mice treated under late prophylactic regimen, five additional mice for each group were considered for immunological studies and for the determination of plasma anti-CII antibodies detection. At the end of the treatment, these mice were sacrificed and spleen and blood were collected.

#### 4.3.1. Preparation of Spleen Cell Suspensions from CIA Mice

Spleens were aseptically removed from 5 mice in the groups treated under late prophylactic regimen, washed with cold PBS, and the tissues were minced. Single-cell suspensions were prepared by passing each spleen through a 70-μm-cell strainer with the end of a 10-mL plastic syringe plunger. Red blood cells in suspension were lysed with Lysing buffer (Sigma Aldrich, Italy). After repeated washes in PBS, the cells were resuspended in triplicate in 96-well plates and 24-well plates (respectively for cell proliferation study and cytokines detection), cultured with RPMI 1640 complete medium, containing 100 U/mL penicillin, 100 μg/mL streptomycin, and 10% fetal bovine serum (FBS). The cells were counted and adjusted to a density of 2 × 10^6^ cells/mL.

#### 4.3.2. Ex Vivo Effects of DAC on Splenic Cytokine Secretion

Splenocyte suspensions at a density of 2 × 10^6^ cells/well were cultured in triplicate to 24-well plates and re-stimulated with 50 μg/mL of bovine type II C (Chondrex, Inc., Redmond, WA, USA), for 48 h at 37 °C in a 5% CO_2_-humidified atmosphere. Cytokine (IL-17, TNF-alpha, INF-γ, IL-10, and IL-4) levels in the culture media supernatants were assayed by ELISA test (Invitrogen, Thermo Scientific Fisher, Milan, Italy), in accordance with manufacturer’s recommendations.

#### 4.3.3. Splenocytes Proliferation, BrdU Incorporation Method

The splenocytes were plated in a 96-well plate at the density of 10 × 10^4^ cells/mL (100 µl/well) and the quantification of the cell proliferation was evaluated by BrdU (Cell Proliferation ELISA, Roche, Mannheim, Germany) incorporation (10 µL/well), 24 h before the end of the 72 h of incubation with CII (Chondrex), as per manufacturer’s instructions. The absorbance of the samples was measured in an ELISA reader at 450 nm.

#### 4.3.4. Anti-CII Antibody Levels

At the end of the study, five mice were sacrificed and sera collected and tested for antibodies directed to bovine native CII by enzyme-linked immunosorbent assays (ELISAs). Plates were coated with CII (Chondrex) in carbonate buffer (pH 9.6) at 4 °C for 16 h. After blocking with 10% BSA (Sigma Aldrich, Milan, Italy) in PBS for 2 h, sera (100 µL) were added to the wells and incubated for 2 h at RT. After washing, biotin conjugated goat anti-mouse total IgG (Thermo Fisher Scientific, Milan, Italy) was added into the plate and incubated for 2 h at RT. After washing, streptavidin-HRP (Sigma Aldrich) was added for 30 min at RT. Then, tetramethylbenzidine (Sigma Aldrich, Milan, Italy) and H_2_O_2_ in citrate phosphate buffer were added to each well. The color reaction was stopped with 1N HCl. The absorbance was measured at 450 nm using a microplate reader.

The results are expressed as absorbance (optical density, O.D.) in experimental wells minus the background absorbance obtained in wells without CII.

#### 4.3.5. Histological Examination

Five additional mice for each group were sacrificed at the end of the late prophylactic treatment with DAC, its vehicle, or dexamethasone. Paws were removed and fixed in 10% formalin for histological examination by an observer unaware of the treatment of the mice. Paws were trimmed placed in decalcifying solution for 24 h, embedded in paraffin, sectioned at 5 μm, stained with hematoxylin and eosin and studied using light microscopy. The following morphologic criteria were used: 0 = no damage; 1 = edema; 2 = presence of inflammatory cells; and 3 = bone erosion [70].

### 4.4. Statistical Analysis

Data are presented as mean ± standard deviation. For the evaluation of statistical significance, the ANOVA test followed by the Fisher’s Least Significant Difference (LSD) test was used.

Differences in disease incidence was evaluated using Kaplan-Meier analysis. Values of *p* < 0.05 were considered statistically significant.

## 5. Conclusions

For the first time, we have provided here preclinical evidence for the possible use of hypo-methylating drugs in RA. The effects of DAC in a murine model of RA entailed a significant amelioration of the clinical condition, along with in vivo and ex vivo modification of the immunological profile of the mice, that exhibited a diminished production of Th1/Th17 cytokines and reduction of anti-type II collagen autoantibodies. The efficacy of DAC to significantly modulate, both in late prophylaxis and therapy regimens, the course of the disease, in a well-established model of RA represents a solid proof-of-concept for the possible design of phase IIa trials on the use of this drug in the clinical setting.

## Figures and Tables

**Table 1:** Predicted autoimmune diseases potentially targeted by DAC.

Disease	Significance (*p* Value)
Allergic Contact Dermatitis	<0.00001
Ankylosing Spondylitis	<0.00001
Asthma	<0.00001
Discoid lupus	<0.00001
Multiple Sclerosis	<0.00001
Rheumatoid Arthritis	<0.00001
Ulcerative Colitis	<0.00001
Crohn’s Disease	0.001701
Atopic Dermatitis	0.130141
Type 1 Diabetes	0.141354
Juvenile Rheumatoid Arthritis	0.423885
Systemic Juvenile Idiopathic Arthritis	0.45051
Systemic Lupus Erythematosus	0.819002
Psoriasis	0.986509
Sjogren’s syndrome	0.989622
Non-Systemic Juvenile Idiopathic Arthritis	0.993984
Dermatomyositis	1
